# NanopoReaTA: a user-friendly tool for nanopore-seq real-time transcriptional analysis

**DOI:** 10.1093/bioinformatics/btad492

**Published:** 2023-08-07

**Authors:** Anna Wierczeiko, Stefan Pastore, Stefan Mündnich, Anne M Busch, Vincent Dietrich, Mark Helm, Tamer Butto, Susanne Gerber

**Affiliations:** Institute of Human Genetics, University Medical Center of the Johannes Gutenberg University Mainz, Mainz 55131, Germany; Institute of Pharmaceutical and Biomedical Sciences, Johannes Gutenberg-University Mainz, Mainz 55128, Germany; Institute of Pharmaceutical and Biomedical Sciences, Johannes Gutenberg-University Mainz, Mainz 55128, Germany; Institute of Human Genetics, University Medical Center of the Johannes Gutenberg University Mainz, Mainz 55131, Germany; Institute of Human Genetics, University Medical Center of the Johannes Gutenberg University Mainz, Mainz 55131, Germany; Institute of Pharmaceutical and Biomedical Sciences, Johannes Gutenberg-University Mainz, Mainz 55128, Germany; Institute of Pharmaceutical and Biomedical Sciences, Johannes Gutenberg-University Mainz, Mainz 55128, Germany; Institute of Human Genetics, University Medical Center of the Johannes Gutenberg University Mainz, Mainz 55131, Germany

## Abstract

**Summary:**

Oxford Nanopore Technologies’ (ONT) sequencing platform offers an excellent opportunity to perform real-time analysis during sequencing. This feature allows for early insights into experimental data and accelerates a potential decision-making process for further analysis, which can be particularly relevant in the clinical context. Although some tools for the real-time analysis of DNA-sequencing data already exist, there is currently no application available for differential transcriptome data analysis designed for scientists or physicians with limited bioinformatics knowledge. Here, we introduce NanopoReaTA, a user-friendly real-time analysis toolbox for RNA-sequencing data from ONT. Sequencing results from a running or finished experiment are processed through an R Shiny-based graphical user interface with an integrated *Nextflow* pipeline for whole transcriptome or gene-specific analyses. NanopoReaTA provides visual snapshots of a sequencing run in progress, thus enabling interactive sequencing and rapid decision making that could also be applied to clinical cases.

**Availability and implementation:**

Github https://github.com/AnWiercze/NanopoReaTA; Zenodo https://doi.org/10.5281/zenodo.8099825.

## 1 Introduction

In standard sequencing experiments, practical steps and data analysis are usually performed independently, with the latter initiated by bioinformatics experts once after sequencing is complete. Nowadays, new technologies such as Oxford Nanopore Technologies (ONT) offer a unique opportunity to start downstream analysis while sequencing is still ongoing (Amarasinghe *et al.* 2020, Wang *et al.* 2012). Some platforms, such as EPI2ME from ONT (https://labs.epi2me.io/) or minoTour (https://github.com/minoTour/minoTour, Munro *et al.* 2022), already provide real-time pipelines for rapid ONT data acquisition integrated into a user interface (UI), and thus accessible to users with limited bioinformatics skills. However, as these platforms’ focus mainly lies on the analysis of DNA-sequencing data, there is a lack of real-time applications in the field of transcriptomics. Here, we introduce NanopoReaTA, an on-demand toolbox for real-time transcriptomic analysis that provides rapid insight on RNA-sequencing data from ONT. Users receive transcriptome-wide and gene-specific information directly while sequencing is still running, such as differences between conditions or expression levels of individual genes. In addition, implemented quality control features allow the user to monitor data variability during the ongoing sequencing process. Ultimately, the tool can provide frequent biologically relevant snapshots of the current sequencing run, which in turn can enable interactive fine-tuning of the sequencing run itself, facilitate decisions to abort the ongoing run to save time and material, e.g. when sufficient accuracy is achieved, or even accelerate the resolution of clinical cases with high urgency.

## 2 Material and methods

### 2.1 Test data

NanopoReaTA has been tested on self-generated direct cDNA-sequencing data from Hek293 and HeLa cells (Supplementary Table S1 and Supplementary Figs S1–S9).

### 2.2 Usage

**Figure 1.**
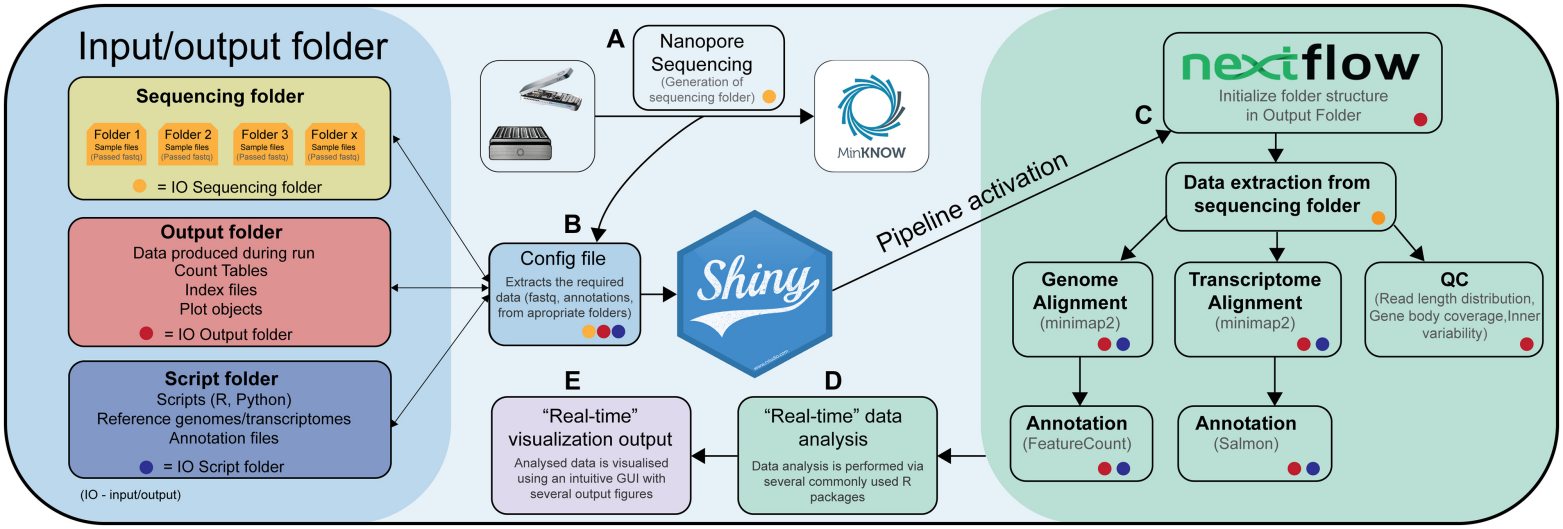
NanopoReaTA workflow visualized as a graphical sketch. (A) Sequencing start; (B) Configuration of settings in NanopoReaTA’s UI; (C) Preprocessing pipeline by *Nextflow;* (D) Transcriptional analysis and (E) visualization of results in NanopoReaTA’s UI. Detailed information on the individual modules is given in the Supplementary Information, as well as in the user manual on the GitHub repository: https://github.com/AnWiercze/NanopoReaTA.

NanopoReaTA can be launched directly after starting a sequencing run of cDNA or direct RNA via ONT’s sequencing software MinKNOW (Fig. 1A and Supplementary Fig. S1). Within NanopoReaTA’s UI, the user will be guided through several configuration settings to extract all information required for data processing such as reference sequences, annotation files, output directory defined in MinKNOW (into which sequencing output is written), and more (Fig. 1B and Supplementary Fig. S2A–C). Preprocessing of basecalled reads from a running or completed experiment is integrated into a *Nextflow* pipeline and can be started via a one-button-click within the UI (Fig. 1C and Supplementary Fig. S2D; Di Tommaso *et al.* 2017). As soon as sequencing data are generated, the *Nextflow* pipeline automatically updates generated files, including gene counts or mapping files. Based on the output files from preprocessing, downstream analyses can be performed within the following tabs integrated into NanopoReaTA: “Overview,” “Gene-wise Analysis,” and “Differential Expression Analysis” (Supplementary Figs S4–S6). The resulting figures can be constantly updated during sequencing (Supplementary Figs S7 and S8). See more details in the Supplementary Information.

### 2.3 Preprocessing via *Nextflow*

The *Nextflow* pipeline takes all fastq files that pass the quality threshold defined in MinKNOW and performs genome and transcriptome alignment using minimap2 (Li 2018) as well as feature quantification using FeatureCounts (Liao *et al.* 2014) and Salmon (Patro *et al.* 2017). In addition, we incorporated a quality control utility extracting sample- and group-wise read length distribution, variability measurements, genome/transcriptome coverage based on RSeQC (Wang *et al.* 2012), and gene count per iteration, enabling the assessment of specific quality metrics over time (Supplementary Fig. S7). See more details in the Supplementary Information.

### 2.4 Downstream analyses based on R

The subsequent downstream analyses are based on commonly used R packages such as DESeq2 (Love *et al.* 2014) for principal component analysis and differential expression analysis of gene and transcript expression, and DEXSeq (Anders *et al.* 2012) and DRIMSeq (Nowicka and Robinson 2016) for differential transcript usage (Fig. 1D and E). In addition, gene body coverage and counts per sample and condition can be visualized for a subset of genes of interest (Fig. 1E). All tables and figures can be downloaded via button clicks (Supplementary Figs S3–S6). See more details in the Supplementary Information.

### 2.5 Installation and requirements

NanopoReaTA can be installed on Linux and Windows via docker by pulling a prebuild docker image containing all package requirements. For installation, requirements, and user manual, visit https://github.com/AnWiercze/NanopoReaTA.

*Hardware:* 64GB RAM, 16 threads. *Software:* Docker.

## 3 Discussion

NanopoReaTA represents a real-time analysis toolbox that allows users to perform interactive transcriptional analyses of cDNA and direct RNA-sequencing data in real-time via a user-friendly and intuitive UI based on R Shiny. We aim to provide a tool that supports users from biological research and clinical diagnostics of transcriptomics by accelerating decision-making processes of future experiments or patient treatment, especially when time and money are limiting factors. For future perspectives, we envision that additional functions such as novel transcript detection, RNA modification detection, and integration of multi-omics levels in real-time can be integrated. NanopoReaTA is open source to also enable the scientific community to contribute such enhancements.

## Supplementary Material

btad492_Supplementary_Data
